# Factors affecting integration of midwifery nursing science theory with clinical practice in Vhembe District, Limpopo Province as perceived by professional midwives

**DOI:** 10.4102/phcfm.v8i2.997

**Published:** 2016-05-24

**Authors:** Thivhulawi Malwela, Sonto M. Maputle, Rachel T. Lebese

**Affiliations:** 1 Department of Advanced Nursing, University of Venda, South Africa

## Abstract

**Background:**

Professional midwives have an important role to play in midwifery training to produce a competent midwife. According to the social learning theory, professional midwives act as role models for students. When allocated for clinical learning experiences in the training hospitals, students will have the opportunity to observe the well-trained, skilled, and experienced professional midwives. The whole process will enable students to integrate theory with practice and they will become competent.

**Aim:**

The aim of this study was to determine the factors affecting integration of midwifery nursing science theory with clinical practice as perceived by midwives.

**Setting:**

The study was conducted at the training hospitals in Vhembe district of the Limpopo Province, South Africa. These hospitals were: Donald Fraser, Siloam, and Tshidzini.

**Methods:**

A qualitative explorative, descriptive and contextual design was used. A Non-probability, convenient sampling method was used to select 11 midwives from the following hospitals: Donald Fraser, Siloam, and Tshidzini, in Vhembe district. In-depth individual interviews were conducted. Data were analysed through open coding method.

**Result:**

One theme and five sub-themes emerged from the analysed data, namely: shortage of midwives, attitudes towards student midwives, reluctance to perform teaching functions, language barriers, and declining midwifery practice standards.

**Conclusion:**

Shortage of midwives in the clinical areas led to fewer numbers of mentors whom the students could observe and imitate to acquire clinical skills. Some of the midwives were reluctant to teach students. Recommendations were made for both training institutions and hospitals to employ preceptors for students in the clinical practical.

## Introduction

Professional midwives have an important role to play in midwifery training to produce a competent midwife. During midwifery training students are taught theory and practice. The theoretical aspects covered would include scope of practice and relevant legislation pertaining to midwifery, low and high risk pregnancy, labour, puerperium, and neonate. Fitzgerald et al.^1^ pointed out that clinical skills underpin midwives’ professional practice, and therefore students need effective opportunities to learn, develop, and master skills related to the theory. According to the social learning theory, professional midwives act as role models for students. When allocated for clinical learning experiences in the training hospitals, students will have the opportunity to observe the well-trained, skilled, and experienced professional midwives. The clinical team should appropriately manage allocations and the workload of team members to assist learning, guide established needs of students, and facilitate the requisite learning.^2^ The whole process will enable students to integrate theory with practice and they will become competent.^3^ Professional midwives have the responsibility to create a psychological climate which is conducive to learning and this will automatically create a safe physical environment for learning.^4^ The safe learning environment will benefit both the student and patient, and will enhance the development of a student to become a safe and accountable practitioner who will add value to the health care service delivery.^5^ The professional midwife needs to allow students to participate actively and enhance professional socialisation, delegate to the students some responsibilities under supervision, and not take all students as irresponsible.^5^ The professional midwife needs to guide students on the procedures in which they are not yet competent; this support will help students to move from the attitude of not knowing, to that of knowing; from not being able to do, to being able to do.^6^ Support roles are undertaken by more experienced, and often more senior registered nurses to assist learning guides. These support roles include clinical teacher, link mentor, clinical facilitator, clinical guide, clinical coach, practice facilitator, practice educator, practice placement facilitator, and clinical education supervisor.^7,8,9^

At first, students may need support to a greater extent, but later they will need less support.^5^ This in turn will facilitate students being involved in their own learning and will make use of clinical learning opportunities offered to them.^10^ However, the integration of theory to clinical practice at the training institution in Vhembe district was not adequately achieved. The factors that affect integration of midwifery nursing science theory with clinical practice are critical and were explored.

## Purpose of the study

The purpose of this article was to determine factors affecting integration of midwifery nursing science theory with clinical practice as perceived by midwives in the selected training hospitals of Vhembe district, Limpopo Province to inform future midwifery training and practice.

## Objectives

Explore the perceptions of midwives toward integration of midwifery theory to clinical practice in the selected training hospitals of Vhembe district, Limpopo Province.

Describe factors affecting integration of midwifery theory to clinical practice in the selected training hospitals of Vhembe district, Limpopo Province.

## Research methods and design

### Study design

A qualitative explorative, descriptive, and contextual design was used to identify and describe factors that affect integration of midwifery theory to clinical practice as perceived by midwives in the training hospitals.

### Study population and sampling strategy

The population consisted of all professional midwives who are allocated in labour wards of the training hospitals in Vhembe district of Limpopo Province. Non-probability, convenient, and purposive sampling was used to select 11 professional midwives from training hospitals, namely; Donald Fraser, Siloam, and Tshidzini. The inclusion criteria were; participants should be a midwife who has been allocated in the labour ward for at least 2 years, and should be willing to share their knowledge and sign an informed consent form.

### Data collection methods

An appointment for interview was secured with participants during lunch hour and/or after work. Data were collected in an office by means of a face-to-face in-depth individual interview.^11^ The interview focused on perceived factors that affect integration of midwifery theory to midwifery clinical practice. The question which guided the interview was *In your opinion what are factors that affect the integration of midwifery theory to midwifery practice during the training of midwifery students?* The question was followed by probing as a communication skill which elicited more information from the participants as postulated by De Vos.^11^ The interviews were conducted in the local language (TshiVenda) by researchers for about 45 min to 1 h. Permission to use a voice recorder was obtained and recordings were transcribed verbatim and translated into English.^6^ Data was collected from participants until data saturation was reached.^12^

### Data analysis

The narrative data from the in-depth interviews were analysed qualitatively using Tesch’s open coding method cited in Botma et al.^13^ The method included the following steps: the researcher read carefully through all the transcripts to get a sense of whole. After the completion of all transcripts, a list of similar topics was compiled. One theme and its sub-themes emerged and field notes were also coded and categorised. A literature control was done to contextualise the results of the study in existing literature.^13,14^

## Results

The results indicating perceived factors that contribute to poor integration of midwifery theory to midwifery practice and sub-themes are presented in Table 1.

**TABLE 1:** Themes and sub-themes.

Theme	Sub-themes
Perceived factors that contribute to poor integration of midwifery theory to midwifery practice	Shortage of midwives
	Attitudes towards student midwives
	Reluctance of midwives to perform teaching function
	Language barrier
	Declining midwifery practice standards

*Source:* Authors own work

In discussing the theme and sub-themes, quotations and relevant literature were used to emphasise the results.

### Theme 1: Perceived factors that contribute to poor integration of midwifery theory to midwifery practice

The discrepancies between theory and practice have been caused largely by the deficiencies in the practice of nursing itself or in the classroom.^15^ The theoretical and practical components of nursing as a profession form the basis of nursing education and require integration of theory and practice. The content covered in the classroom should relate to the experiences of the student nurses in the real clinical setting. The theme that emerged from data analysis was perceived factors that contribute to poor integration of midwifery nursing science theory with clinical practice. Sub-themes that emerged from this theme were shortage of midwives, attitudes towards student midwives, reluctance to take their teaching function, language barriers, and declining midwifery practice standards. These sub-themes will be discussed individually.

#### Sub-Theme 1: Shortage of Midwives

Shortage of midwives was described as less than the ideal number of midwife practitioners relative to the number of students in a teaching hospital unit. In this study, it was revealed that midwives also had a negative perception of and attitude towards students. The reluctance and unwillingness of midwives to perform their teaching functions, for example to mentor or demonstrate procedures to students, have become major stumbling blocks in the provision of efficient nursing and midwifery training. The quote from one participant was:

‘Ok, shortage of staff, we have got few midwives who need to attend to students as well as patients. There is lack of accompaniment by lecturers, sometimes you find that students need to be taught certain procedures and due to this shortage of staff we do not manage to teach them, we will just do a summary.’ (Participant 8)

Midwives indicated that even if 3 midwives were on duty with 12 students, the latter would be outnumbering the midwives on duty for that day. The same midwives were expected to provide care to pregnant and postnatal women and their newborn babies. This made it difficult for the midwives to execute their teaching role; they ended up helping the mothers to avoid loss of life. The units needed to have sufficient numbers of midwives to cover for the needs of patients and those of the students. This, coupled with the language barriers imposed by midwives when they communicate to students in the vernacular instead of the English (academic) language, as well as non-adherence to midwifery practice standards and norms, continues to compromise nursing and midwifery education and practice.

#### Sub-Theme 2: Attitude towards Student Midwives

The midwives indicated the factor of negative attitudes of qualified midwives toward students. These midwives had a negative attitude towards teaching students. Some midwives developed the negative attitude because of problems they encountered with previous groups of students. Some midwives labelled the comprehensive (R.425) 4-year students as lazy and irresponsible.^16^ This was supported by the following quote:

‘Another issue is the attitude of the midwives towards students and visa versa. Therefore, if I have identified that student so and so as a dodger I will have a negative attitude to her and this jeopardizes her learning, the other thing is that some procedures will be done when she is absent and she will never know because (*o vha a siho*) she was not there.’ (Participant 3)

One participant indicated that some midwives had an attitude towards the comprehensive (R.425) students because they would be more qualified at the end while they themselves were doubly qualified. The quote cited was:

‘Some like us bar one becomes jealous because these students will come out with three bars.’ (Participant 1)

The other contributory factor was that college students received stipends and they were perceived as being lazy and they tended to be treated differently. Because of the stipend, students were taken as workforce and not considered as students who should be guided to acquire midwifery skills.

#### Sub-Theme 3: Reluctance of Midwives to Perform Teaching Function

The respondents indicated that as midwives they were not keen to teach, some said they were not lecturers, while others lacked the skills to teach. Some midwives went to the extent of telling students that they are not lecturers, and students were expected to wait for their lecturers to come and teach them. The following quote was cited:

‘This ward is so busy, and there is a shortage of staff and I don’t have time to teach the students. The lecturers must also make accompaniment to evaluate them.’ (Participant 11)

One midwife participant said:

‘Even if you have time to teach, students will be allocated to the ward without objectives. So what will I teach them.’ (Participant 5)

The midwives participants felt that staff members should be complimented and or the lectures should do accompaniment for the students. However, it was noted that when students were in the level 4 as finalists; midwives did not supervise them; they would leave everything upon them because they were viewed as their colleagues now and students could do things on their own. This was not appropriate because students needed their support until they became qualified; even after qualifying they would still need support from the experienced midwives. Longworth^2^ pointed out that support roles are undertaken by more experienced, and often more senior registered nurses to assist learning guides. To enable students to continue to link theory to practice, mentors must demonstrate the knowledge and skills required and enable opportunities for students to practice their skills to promote theory-practice assimilation and thus enhance student education.

#### Sub-Theme 4: Language Barrier

A language barrier was found to affect integration of theory to practice. Participants indicated that some qualified midwives were using their own language during report giving; this made students not learn as they were all not Vendas or Sothos. One participant supported the issue of language problems in a training hospital; midwives were using their native language when they are executing duties in the units. The participants further emphasised that it was a must to use English, more especially in training hospitals because English was their medium of instruction during training. This was supported by the following quote:

Some other problem is language you will find *“uri”* (it means) students learn in English, I know in RSA is democracy, but when they get to the hospital they have to grab complicated words in Venda words, so that thing discourages them to learn, some of the words they can’t hear properly as they are not Venda speaking. I am not a Venda or English, the two languages make things complicated. At the hospital they perform procedures with a Venda person it is difficult to correlate theory with practice.

Another participant supported this between supported and when stating her experience by saying:

‘Yes is true, I am from advanced midwifery training at a tertiary hospital, there they will be using their language (Sepedi) in everything. As we are from Venda …we were not learning much, except in the class for theory. This was very discouraging as you will be afraid to ask certain things because they will be amazed. English should be used as a medium of instruction.’

#### Sub-Theme 5: Declining Midwifery Practice Standards

Practicing midwives are no longer adhering to the midwifery practice that was taught during training. The participants indicated that some midwife practitioners were no longer committed to their practice; they usually overlooked issues of maintaining sterility during internal examinations and other procedures that needed sterility. They no longer set trolleys for procedures, and did not follow some steps in their daily routine care, for example, washing of hands in between patients. They also no longer took their responsibility to supervise students in the clinical settings. According to social learning theory, learning occurs through observation; students learn by observing practitioners who are experts in the field of midwifery. If the standards were declining, proficiency by the midwife would be minimal, interrupting the vicarious reinforcement that learning happens by imitating others.^3^ Participants complained of the shortage of medical equipment and supplies as another factor that put the training of midwives at risk. There were shortages of gloves, per vaginal examination packs, and other medical supplies. When there are no gloves you cannot teach procedures like per vaginal examination, suturing of episiotomy, and many others, in fact in maternity section gloves are priority because we are dealing with human blood and other body fluids. The absence of packs makes it difficult to demonstrate sterile and other surgical clean procedures. All this compromised the training of students, because the integration of theory to practice was not achieved. Participant said:

‘As far as equipments are concerned we are having a problem, we do not have enough equipments, more especially when we come to suturing or stich packs, you will find that in this situation now we are not having stich packs. Sometimes you may find that inside the delivery packs there is no needle holder, when you teach the student you will find that there is an artery forceps, it will need somebody who is experienced to suture. You may find that there is no delivery pack you will need to use a PV pack to deliver patients. PV packs does not have all equipment for delivery.’ (Participant 7)

She continued by saying:

‘Yes just like some month back we were not having the cord clamps, and you find ‘uri’ that we are using tags or ligatures to tie the newborns cords and this is very difficult when expected to teach students because we ourselves are not used to this tags , so when it comes to students it becomes very tough.’ (Participant 7)

Lack of necessary equipment interfered with the integration of theory to practice. In support of what the respondents said, the National perinatal morbidity and mortality committee report 2008–2010 outlined the report on the survey of equipment, which was done by Department of Health^17^ in the neonatal units of South Africa. The report showed that 64 district hospitals accounting for 68% of total hospitals lacked radiant warmers, 63 lacked resuscitation equipment, 19 had no suction machines, and 82 lacked equipment to monitor vital signs. The shortage of equipment affected training negatively as students would not get opportunities to observe necessary procedures as there was no equipment to perform such procedures. Midwives did not execute their duties procedurally when there were no gloves, thus interfering with their role modelling and mentoring of student midwives.^17^ The training institution should be provided with training equipment. The literature supports the notion that a professional nurse or midwife should help a student to make things happen and to diagnose, plan, implement, and evaluate systematically. The professional nurse or midwife smooths problems encountered by calling upon experience, contacts, and extra resources. She sets standards and criteria that are to be maintained to help students to focus within each topic or subject in order to merge theory with practice. She sets outcomes that are measurable, and assessment tools, which will help students to meet their training needs.^18^

### Trustworthiness

The criteria for ensuring trustworthiness as outlined by Lincoln,^19^ were observed. Credibility was ensured by prolonged engagement in order to build trusting relationships with the participants.^11^ The researchers had contact with participants during the appointment-making session, the information session, and during data collection. Referential adequacy was achieved by taking notes to record findings that provided a suitable record, and the use of a voice recorder. Transferability was ensured by thick descriptions of research methodology. Member check was also conducted in order to validate the truth and to confirm the results.^19^

### Ethical considerations

Researchers obtained the approval from the University of Venda (UNIVEN) ethical committee. The ethical clearance project no: SHS/13/PDC/07/1605 was obtained from the UNIVEN ethical committee. Limpopo Provincial Department of Health granted a letter for permission to access facilities and collect data from the training hospitals. Informed consent was also obtained from each participant.

## Discussion

The findings of the study indicated that there was a shortage of midwives in all three training hospitals which made it difficult for midwives to perform their dual roles of patient care and that of teaching the students. The shortage of midwives led students to take shortcuts in most of the procedures. Such practices can defame their role-modelling image, and cause students to learn wrong principles and methods of procedures, hence interfering with successful integration of theory with practice. The shortage of midwives in the unit was noted as a factor that interrupted the proper integration of midwifery nursing science theory into clinical practice. Midwives in students’ training are key facilitators in influencing the learning environment in hospital placement. They also serve as a role model for nursing practice. Neary^18^ was of the notion that professional nurse manager’s style and personality are also important in determining effective learning environment.^11^ Some midwives developed the negative attitude because of problems they encountered with previous groups of students. Some midwives labelled the comprehensive (R.425) 4-year students as lazy and irresponsible. Midwives also had negative attitudes towards students because they perceived that as they receive stipends, they should become part of the workforce and not students who needed their assistance. Some participants went to an extent of telling students that: ‘I am not a lecturer, wait for your lecturers to come they should teach you’.

Midwives must treat students with kindness and understanding; try to show interest in students as people. They should take students as learners, not extra pairs of hands, and foster their self-esteem; make the students to feel as part of the team.^20^ Midwives may act as a mentor and/or preceptor who guides the students towards an establishment place in the midwifery profession.^21^ They can support the student taking the pre-registration nursing course or guide the student through many hours of clinical or practical work and keep in contact with the tutor in order to forward the student progress.^18^

Midwives in the units were using their native language to communicate when giving report. Kocakulah, Ustunluoglu^22^ was of the opinion that if students are exposed to everyday concepts by using their native language, it will be easier for them to understand scientific concepts in a classroom setting, however, in this context, this led to missed learning opportunities because some students were from different regions of the Limpopo Province and were not acquainted with the used native language. The use of English as an official language in South Africa is recommended.

The standards included delegating students according to their level of competence and supervising or supporting them.^23^ The midwives were expected to provide a high standard of practice and care at all times, to keep their skills and knowledge up-to-date, not to be partial, to uphold the reputation of their profession, and to act with integrity. All these standards were meant to facilitate excellent patient care and thus assist the students in the placement to learn by imitation and observation.^24^ Declining midwifery standards meant that the explained standards were low, distorting the image of midwifery practice and thus made students observe and learn poor midwifery care. Lack of equipment was a serious issue at training institutions. A study done in Pakistan indicated that government hospitals lacked resources as well as the required number of doctors. Hospitals where doctors were present were helpless because of the absence of proper resources to treat their patients. Those who cannot afford private doctors wait for death to ease their suffering. Most of the health care facilities in such areas were provided by non-government organisation (NGO) run hospitals and clinics because of the lack of resources. Lack of material also affected training of health workers as there would be no equipment to show students practitioners correct procedures. Students ended witnessing wrongly performed procedures affecting their chance to put theory into practice.^25^

According to the social learning theory used in this study, the professional midwives should act as role models.^26^ The midwife practitioners needed to adhere to midwifery care standards to provide competent, proficient midwifery care. The students were motivated to learn when they saw a professional who was skilled and competent enough to provide excellent and accurate care.^3^ Failure to adhere to midwifery standards deprives students of the opportunity to learn by observation and imitating the midwives.

## Recommendations

Training hospitals should employ more staff to cater to the needs of both patients and students. The training hospitals need to include the role of teaching and modelling in the orientation programme for all newly employed midwives and conduct regular in-service training on their roles as teachers and models towards midwifery students. Unit managers need to devise teaching programmes in consultation with midwives and students and make sure these are implemented. The unit manager and the college management need to develop guidelines that would allow midwives to discipline students who misbehave in the clinical placement, for example not to sign for procedures that the student did not perform or make the student time off if need be. Midwives need the support of supervisors and courses on stress management techniques to reduce stress and promote a good working environment that is conducive for learning. Midwives in the units need to treat students as unique and not generalise or judge all students on the basis of a mistake of one student. Midwives need to use the official medium of instruction, which is English, to facilitate learning during report giving. Midwives need to involve themselves in personal development and growth to become familiar with contemporary issues in midwifery to boost their self-esteem and ability to assist students with confidence.

## Conclusion

The results of the study revealed various factors affecting integration of midwifery nursing science theory with clinical practice as perceived by midwives in selected training hospitals of Vhembe District, Limpopo Province. The results also indicated that there was poor integration of midwifery nursing science theory with clinical practice in the Vhembe District.
